# Evaluation of plan robustness on the dosimetry of volumetric arc radiotherapy (VMAT) with set-up uncertainty in Nasopharyngeal carcinoma (NPC) radiotherapy

**DOI:** 10.1186/s13014-021-01970-8

**Published:** 2022-01-03

**Authors:** Zhen Ding, Xiaoyong Xiang, Qi Zeng, Jun Ma, Zhitao Dai, Kailian Kang, Suyan Bi

**Affiliations:** Department of Radiation Oncology, National Cancer Center/National Clinical Research Center for Cancer/Cancer Hospital and Shenzhen Hospital, Chinese Academy of Medical Sciences and Peking Union Medical College, No. 113 Baohe Rd, Longgang District, Shenzhen City, 518116 Guangdong Province People’s Republic of China

**Keywords:** Robustness, Tumor control probability, Normal tissue complication probability, Set-up uncertainty

## Abstract

**Purpose:**

To evaluate the sensitivity to set up the uncertainty of VMAT plans in Nasopharyngeal carcinoma (NPC) treatment by proposing a plan robustness evaluation method.

**Methods:**

10 patients were selected for this study. A 2-arc volumetric-modulated arc therapy (VMAT) plan was generated for each patient using Varian Eclipse (13.6 Version) treatment planning system (TPS). 5 uncertainty plans (U-plans) were recalculated based on the first 5 times set-up errors acquired from cone-beam computer tomography (CBCT). The dose differences of the original plan and perturbed plan corresponded to the plan robustness for the structure. Tumor control probability (TCP) and normal tissues complication probability (NTCP) were calculated for biological evaluation.

**Results:**

The mean dose differences of D_98%_ and D_95%_ (ΔD_98%_ and ΔD_95%_) of PTVp were respectively 3.30 Gy and 2.02 Gy. The ΔD_98%_ and ΔD_95%_ of CTVp were 1.12 Gy and 0.58 Gy. The ΔD_98%_ and ΔD_95%_ of CTVn were 1.39 Gy and 1.03 Gy, distinctively lower than those in PTVn (2.8 Gy and 2.0 Gy). The CTV-to-PTV margin increased the robustness of CTVs. The ΔD_98%_ and ΔD_95%_ of GTVp were 0.56 Gy and 0.33 Gy. GTVn exhibited strong robustness with little variation of D_98%_ (0.64 Gy) and D_95%_ (0.39 Gy). No marked mean dose variations of D_mean_ were seen. The mean reduction of TCP (ΔTCP) in GTVp and CTVp were respectively 0.4% and 0.3%. The mean ΔTCPs of GTVn and CTVn were 0.92% and 1.3% respectively. The CTV exhibited the largest ΔTCP (2.2%). In OARs, the brain stem exhibited weak robustness due to their locations in the vicinity of PTV. Bilateral parotid glands were sensitive to set-up uncertainty with a mean reduction of NTCP (ΔNTCP) of 6.17% (left) and 7.70% (right). The D_max_ of optical nerves and lens varied slightly.

**Conclusion:**

VMAT plans had a strong sensitivity to set-up uncertainty in NPC radiotherapy, with increasing risk of underdose of tumor and overdose of vicinal OARs. We proposed an effective method to evaluate the plan robustness of VMAT plans. Plan robustness and complexity should be taken into account in photon radiotherapy.

## Introduction

Radiotherapy (RT) is the main strategy for Nasopharyngeal carcinoma (NPC) [1]. Owing to large irradiation volumes, complex and intricate anatomical structures, precision dose coverage, and organ at risk (OAR) sparing were crucial in NPC radiotherapy [2]. Volumetric arc radiotherapy (VMAT) had been widely used in NPC radiotherapy, for VMAT performed optimized dose distribution and OAR sparing by continuous variation of multi machine parameters [3]. However, increased plan complexity elevated risk of dose calculation and delivery, for more complex plans required smaller and irregular beam apertures, larger tongue-and groove effects, and greater extent modulation of machine parameters, including gantry rotation speed, dose rate, and multi leaf collimator (MLC) position [4, 5]. VMAT plans may show the sensitivity of dose delivery to subtle deviations, including machine parameters and target motion [4, 6].

Image guidance, such as the cone-beam computed tomography (CBCT) has been widely used in position verification to reduce patient set-up uncertainty [7]. The protocol of imaging frequency varies among centers to balance the treatment efficiency and accuracy. The unknown remaining fractions may result in unexpected dose deviation and potential tumor recurrence [8]. For this purpose, we aimed to study the sensitivity of highly optimized VMAT plans to geometry deviation to make a more complete description of dose delivery for complex plans.

Treatment plan robustness is the degree of resiliency of the required dose distribution to these uncertainties and varies with the treatment site, technique, and method. Yock’s [9] report reviewed robustness analysis methods and their dosimetric effects, to promote reliable plan evaluation and dose reporting, particularly during clinical trials conducted across institutions and treatment modalities. The concept of robustness had been widely used in proton treatment plans for the sharp distal fall-off and scattering characteristics but was ignored in photon radiotherapy [5]. We adopted a plan robustness quantification method to address the sensitivity of VMAT plans to geometric uncertainty based on the daily CBCT shifts. Besides, the tumor control probability (TCP) and normal tissues complication probability (NTCP) models were applied to evaluate the potential biological dose differences.

## Methods

### Patient selection and delineation

We retrospectively evaluated treatment plans for 10 NPC patients treated in our center. The clinical characteristics of the patients enrolled in this study were shown in Table 1. All the patients were immobilized by a thermoplastic mask in a supine position. The CT image with a 2.5 mm slice thickness was acquired using a 16-slice CT scanner (GE Discovery RT, GE Healthcare, Chicago, IL, USA). The target volumes and organs at risk (OARs) were delineated by the same clinician. The gross tumor volume (GTV) consisted of GTV of the primary (GTVp) and GTV of lymph nodes (GTVn). The clinical target volume (CTV) consisted of CTVp and CTVn. The planned target volume (PTV) included PTVp, PTVn, and PTV. All the GTVs, CTVs, and PTVs were contoured by the same oncologist based on international guidelines [10].

**Table 1:** Patients characteristics

Patient #	Age	Sex	Stage
1	57	Male	T1N1M0
2	62	Male	T4N2M0
3	68	Male	T3N2M0
4	27	Male	T1N2M0
5	28	Female	T1N1M0
6	68	Male	T1N1M0
7	67	Male	T2N2M0
8	43	Male	T3N0M0
9	54	Male	T1N1M0
10	38	Male	T1N1M0

### Treatment plans and uncertainty plans

A 2-arc volumetric-modulated arc therapy (VMAT) plan was generated for each patient using Varian Eclipse (13.6 Version,) treatment planning system (TPS) modeled for the VitalBeam (Varian, Palo Alto, US) linac. Arc 1 (A1) rotate clockwise from 181° to 179°, and the arc 2 (A2) rotates counterclockwise from 179° to 181°. Collimator angles were set at ± 10°. The prescription doses of PTVp, PTVn, and PTV were 69.96 Gy, 68.31 Gy, and 59.40 Gy in 33 fractions, respectively.

**Fig. 1:**
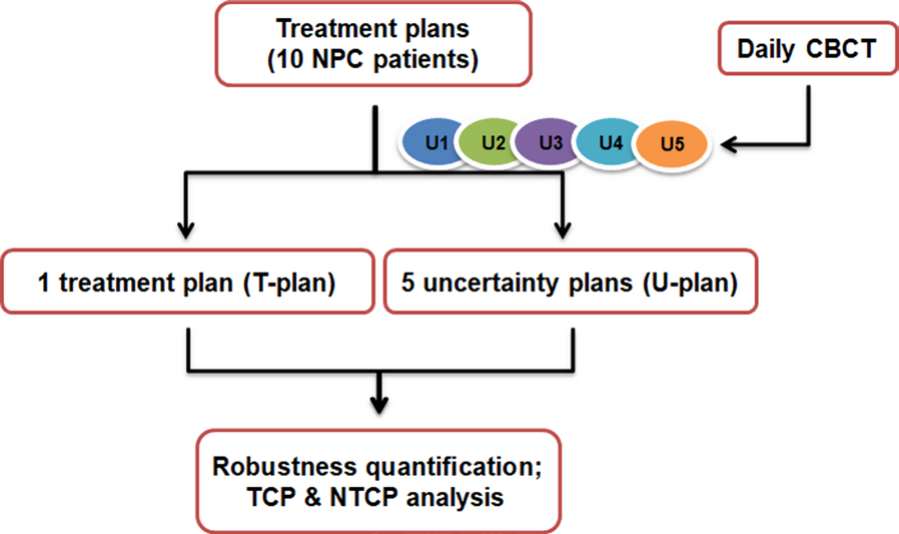
Steps in robustness and biological evaluation of volumetric arc radiotherapy (VMAT) of NPC. U1 ~ U5 represented 5 set-up uncertainties acquired from the first 5 times daily CBCT. T-plan: Treatment plan; U-plan: Uncertainty plan

5 set-up uncertainties were introduced on the original VMAT plan, shifting the isocenter from its reference position according to the set-up errors acquired by CBCT. The U-plans, representing the perturbed plans introduced set-up uncertainties, were calculated for 33 fractions to facilitate the dose comparison. The evaluated items of PTVs and OARs were listed in Table 2 (Fig. 1).

**Table 2:** Evaluated items of PTVs and OARs

PTVs/OARs	Evaluated items
PTVp, PTVn, PTV	ΔD_2cc_, ΔD_98%_, ΔD_95%_, ΔD_mean,_ ΔTCP
CTVp, CTVn, CTV	
GTVp, GTVn	
Brain Stem, Brain Stem PRV	ΔD_max,_ ΔNTCP
Spinal Cord, Spinal Cord PRV	
Lens L, Lens R	
Optic Nerve L, Optic Nerve R, Optic Chiasma	
Parotid L, Parotid R	ΔD_mean,_ ΔNTCP

PTV, planning target volume; PTVp, planning target volume of GTVp; PTVn, planning target volume of GTVn; CTV, clinical target volume; CTVp, primary tumor sites and their invasion range; CTVn, the clinical target volume of GTVn; GTVp, the clinical target volume of GTVp; GTVn, cervical metastatic lymph node. D_mean_ and D_max_ represented the mean dose and the maximum dose. D_x%_ represented the dose (in Gy) received by x% of the volume, V_y Gy_ the volume (in percentage) received by y Gy. D_2cc_ the dose (in Gy) received by a volume of 2 cm^3^

### Robustness quantification method

There are 1 treatment plan (T-plan) and 5 uncertainty plans (U-plans) for each patient. The dose values in the treatment and perturbed plans were displayed in the dose-volume histogram (DVH) curves. D_x%_ represented the dose (in Gy) received by x% of the volume. D_2cc_ the dose (in Gy) received by a volume of 2 cm^3^. D_max_ and D_mean_ represented the maximum and mean dose (in Gy). Absolute differences ΔD, which could be calculated by the absolute value of the minimum value subtracted from the maximum value and corresponded to the plan robustness for the structure.

### TCP and NTCP evaluation

Biological models have been proposed to predict radiobiological response to dose after irradiation [11, 12]. The TCP and NTCP values were calculated to evaluate the biological effects. We use the Schultheiss logit model proposed by Niemierko [13]. We calculated the TCP according to Eq. (1) with the parameters: TCD_50_ = 61.59 Gy, γ_50_ = 3.38 [14].

1 $$TCP = \frac{1}{{1 + \left( {\frac{{TCD_{50} }}{EUD}} \right)^{{4\gamma_{50} }} }}$$

TCD_50_ is the dose of radiation that locally controls 50% of tumors. The γ_50_ is the change in TCP expected because of a 1% change in dose about the TCD_50_. We calculated the NTCP [14] according to Eq. (2)

2 $$NTCP = \frac{1}{{\sigma \sqrt {2\pi } }}\mathop \smallint \limits_{ - \infty }^{EUD} e^{{ - \left( {\frac{{\left( {{\text{x}} - TD_{50} } \right)^{2} }}{{2\sigma^{2} }}} \right)}} dx$$

The σ was calculated by Eq. (3)

3 $${\upsigma } = {\text{m}}TD_{50}$$

The EUD, representing equivalent uniform dose, was calculated according to Eq. (4)

4 $$EUD = \left( {\frac{{\mathop \sum \nolimits_{i} V_{i} D_{i}^{{{\raise0.7ex\hbox{$1$} \!\mathord{\left/ {\vphantom {1 n}}\right.\kern-\nulldelimiterspace} \!\lower0.7ex\hbox{$n$}}}} }}{{\mathop \sum \nolimits_{i} V_{i} }}} \right)^{n}$$

TD_50_ is the tolerance dose yielding a 50% complication rate in the normal organ. V_i_ is the volume at dose D_i_. Parameter m and n are specific dose–response constants [15].

### Statistical analysis

There are 1 T-plan and 5 U-plans for each patient. The dose differences were calculated by the absolute value of the minimum value subtracted from the maximum value and were explicit by mean value (minimum value to maximum value). The dose deviations of D_95%_, D_98%_, D_2cc_, and D_mean_ of CTVs, GTVs, and PTVs were chosen. D_max_ was chosen for serial OARs and D_mean_ for the bilateral parotid gland. The TCP and NTCP reduction were calculated.

## Results

### Targets dose coverage

**Fig. 2:**
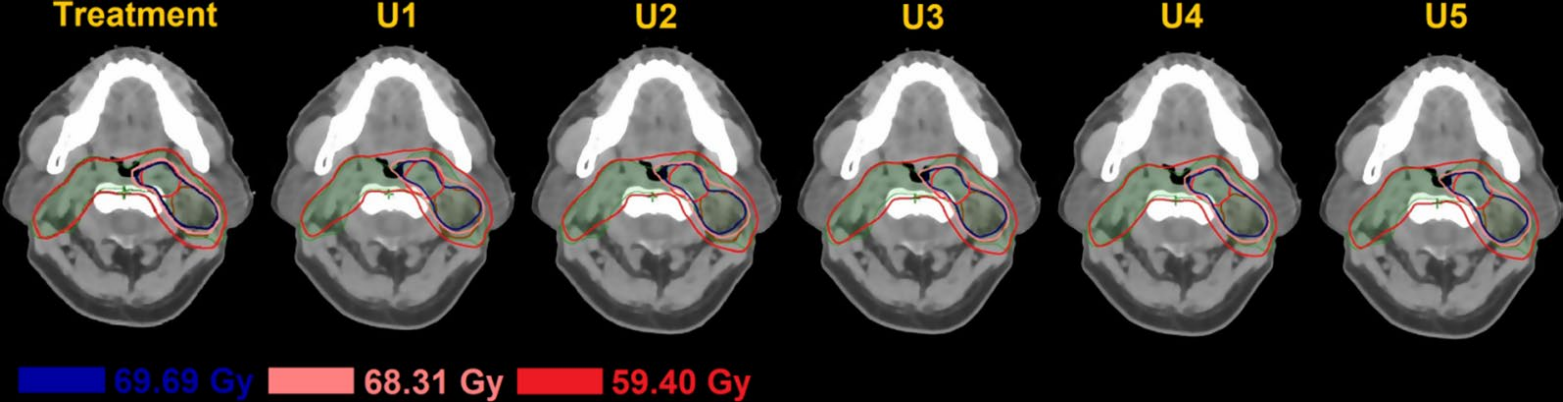
A schematic of transversal dose distributions in 1 treatment plan and 5 U-plans. The green volume represents PTV, the brown volume represents PTVn, and the red volume represents PTVp. The transversal dose coverage varies due to set-up uncertainty

**Fig. 3:**
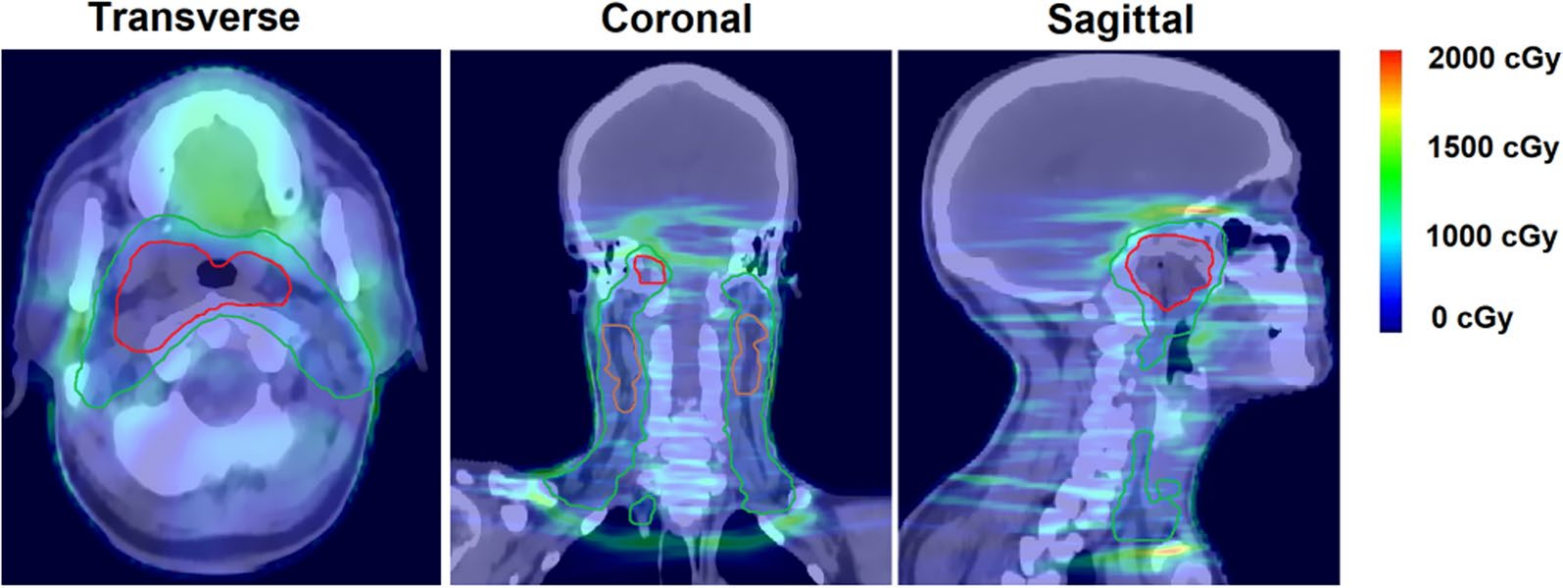
An example of color wash schematic of dose difference bewteen the T-plan and one of the U-plan. The green, brown, and red volumes represent PTV, PTVn, and PTVp respectively

Figure 2 shows a schematic of transversal dose distributions in 1 treatment plan and 5 U-plans. The transversal dose coverage varies due to set-up uncertainty. To visualize the dose difference, a color wash schematic of differences in dose distributions is shown in Fig. 3. The maximum dose discrepancies were observed in marginal zones of PTVs. The dose changes of OARs were also greater in the vicinity of marginal zones and lesser distal to these areas.

The average dose difference was shown in Table 3. No obvious differences were found in D_2cc._ The mean dose differences of D_98%_ and D_95%_ of PTVp were respectively 3.30 Gy and 2.02 Gy. Decreased ΔD_98%_ (1.12 Gy) and ΔD_95%_ (0.58 Gy) were seen in CTVp. The ΔD_98%_ and ΔD_95%_ in GTVp were 0.56 Gy and 0.33 Gy, indicating that the CTV-to-PTV margin promoted the robustness of GTV and CTV. Similarly, the PTVn had the largest difference of D_98%_ (2.77 Gy) and D_95%_ (2.00 Gy). The ΔD_98%_ and ΔD_95%_ of CTVn were 1.39 Gy and1.03 Gy. Minor dose differences were observed in GTVn for both D_98%_ (0.64 Gy) and D_95%_ (0.59 Gy). No marked mean dose variations of D_mean_ were seen. Superior robustness in PTV and CTV was seen.

**Table 3:** Dose difference of PTVs, CTVs, and GTVs in 10 patients

Targets	ΔD_2cc_ (Gy)	ΔD_98%_ (Gy)	ΔD_95%_ (Gy)	ΔD_mean_ (Gy)
PTVp	0.23 (0.07–0.34)	3.30 (0.76–4.38)	2.02 (0.53–3.23)	0.31 (0.11–0.70)
PTVn	0.36 (0.05–0.86)	2.77 (0.60–6.05)	2.00 (0.72–4.56)	0.65 (0.10–1.52)
PTV	0.18 (0.03–0.35)	1.95 (0.26–3.20)	1.34 (0.27–2.01)	0.35 (0.05–0.57)
CTVp	0.20 (0.04–0.35)	1.12 (0.38–3.85)	0.58 (0.23–1.50)	0.16 (0.05–0.30)
CTVn	0.40 (0.08–0.75)	1.39 (0.17–5.81)	1.03 (0.43–4.10)	0.56 (0.12–1.43)
CTV	0.19 (0.03–0.34)	1.17 (0.12–2.44)	0.72 (0.11–1.51)	0.28 (0.06–0.46)
GTVp	0.28 (0.12–0.37)	0.56 (0.06–2.88)	0.33 (0.07–0.67)	0.22 (0.03–0.49)
GTVn	0.39 (00.16–0.74)	0.64 (0.30–1.91)	0.59 (0.16–1.59)	0.44 (0.08–0.84)

The results were exhibited by Mean (Minimum–Maximum)

Table 4 showed the dose differences of OARs. The ΔD_max_ of the brain stem and PRV were 4.34 Gy (1.50 Gy-11.10 Gy) and 6.21 Gy (2.40 Gy-10.19 Gy). The ΔD_max_ of the spinal cord and PRV were 2.86 Gy (1.00 Gy-7.10 Gy) and 3.64 Gy (1.70 Gy-7.40 Gy). Narrowed width of DVH bands was observed in the bilateral lens. Optical nerves performed marked dose difference of mean dose, which were 8.00 Gy, 8.66 Gy, and 8.81 Gy for optical nerve L,R, and chiasma. The D_mean_ of bilateral parotid glands exhibited obvious changes.

**Table 4:** Dose difference of D_max_ or D_mean_ in OARs in 10 patients

Items	OARs	Dose (Gy)
ΔD_max_	Brain Stem	4.34 (1.50–11.10)
	Brain Stem PRV	6.21 (2.40–10.19)
	Spinal Cord	2.86 (1.00–7.10)
	Spinal Cord PRV	3.54 (1.70–7.40)
	Lens L	0.89 (0.20–2.80)
	Lens R	0.79 (0.20–1.40)
	Optical Nerve L	8.00 (1.80–17.40)
	Optical Nerve R	8.66 (1.40–20.20)
	Optic Chiasma	8.81 (1.80–20.80)
ΔD_mean_	Parotid L	4.48 (2.47–7.28)
	Parotid R	4.05 (2.01–6.00)

The results were exhibited by Mean (Minimum–Maximum)

**Fig. 4:**
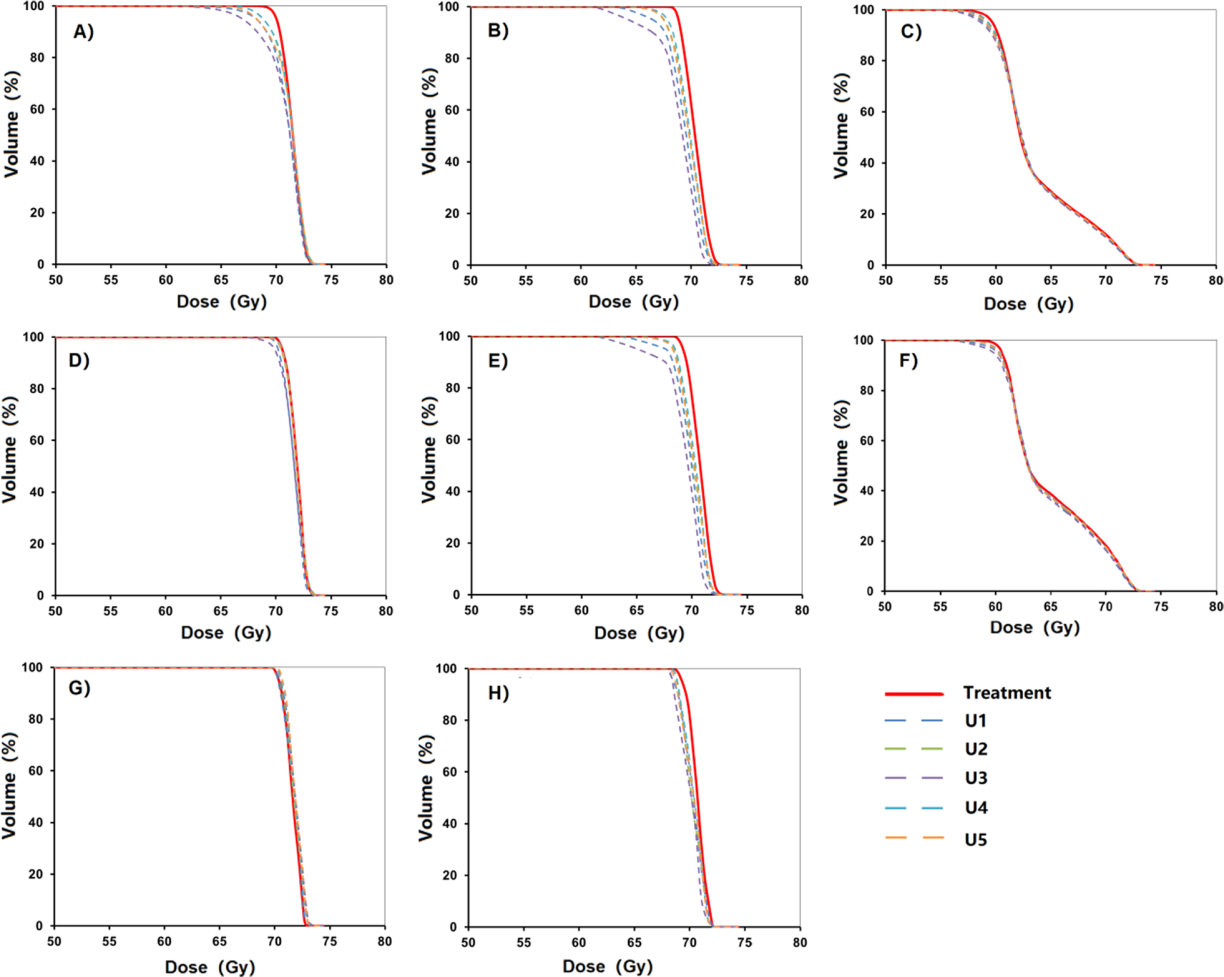
A sample of dose-volume histograms (DVHs) of PTVs, CTVs, and GTVs, compared for 1 treatment plan and 5 U-plans. The DVH curve in the solid line represented the treatment plan. The DVH curves in the dashed line represented 5 U-plans. **A** PTVp; **B** PTVn; **C** PTV; **D** CTVp; **E** CTVn; **F** CTV; **G** GTVn; **H** GTVp

A sample of dose-volume histograms (DVHs) of PTVs, CTVs, and GTVs was shown in Fig. 4. The solid line represented the DVH of the treatment plan, and the 5 dashed lines represented the DVH of U-plans. The envelope was defined as the area between all the DVH curves. The gradually narrowed envelope was seen in PTVp (Fig. 4A), CTVp (Fig. 4D), and GTVp (Fig. 4G). PTVn (Fig. 4B) exhibited high sensitivity to set-up uncertainty. Narrowed width of the envelope was seen in CTVn (Fig. 4E). Sufficient dose coverage and decreased robustness were noticed in GTVn (Fig. 4H). Superior robustness was seen in PTV (Fig. 4C) and CTV (Fig. 4F).

**Fig. 5:**
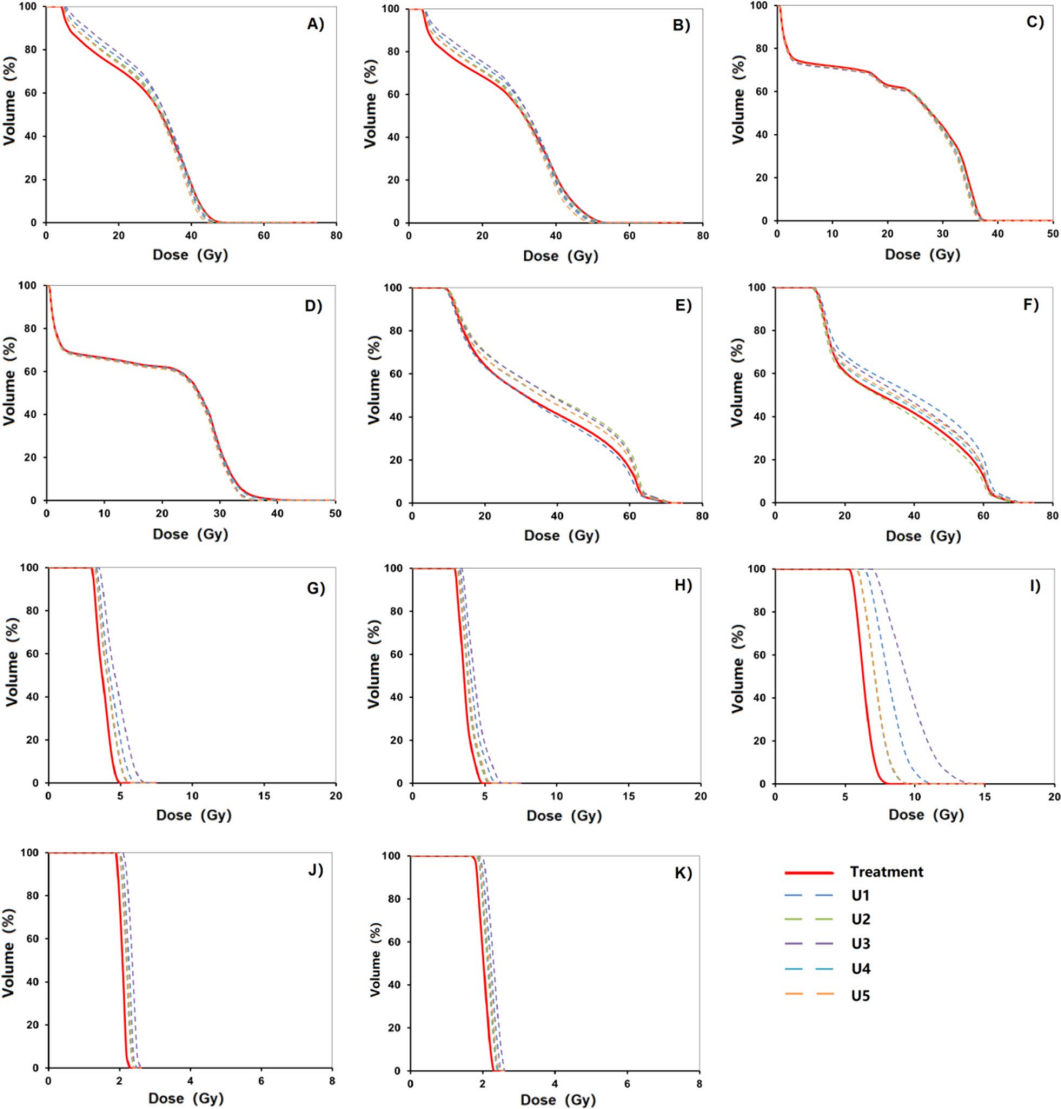
A sample of dose-volume histograms (DVHs) of OARs was compared for 1 treatment plan and 5 U-plans. The DVH curve in the solid line represented the treatment plan. The DVH curves in the dashed line represented 5 U-plans. **A** Brain Stem; **B** Brain Stem PRV; **C** Spinal Cord; **D** Spinal Cord PRV; **E** Parotid L; **F** Parotid R; **G** Optical Nerve L; **H** Optical Nerve R; **I** Optical Nerve Chimsa; **J** Lens L; **K** Lens R

As to OARs (Fig. 5), the brain stem (Fig. 5A) and its PRV (Fig. 5B) exhibited weak robustness due to their locations in the vicinity of PTVs. The spinal cord (Fig. 5C) and its PRV (Fig. 5D) had stronger robustness. Bilateral parotid glands (Fig. 5E. F) were sensitive to set-up uncertainty for their being partially enclosed PTVs. The D_max_ of bilateral optical nerves (Fig. 5G–I) and lens(Fig. 5J, K) varied slightly.

### TCP and NTCP evaluation

**Fig. 6:**
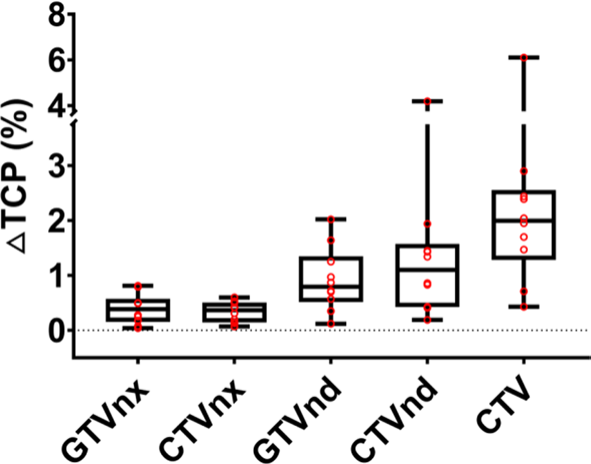
Box plot showed the ΔTCP of all targets due to set-up uncertainties. The ΔTCP was the mean reduction of TCP

The TCP reduction (ΔTCP) was the mean absolute value of the minimum value subtracted from the maximum value. For GTVp and CTVp, the ΔTCP value was less than 1% (Fig. 6), indicating strong robustness to set-up uncertainty. A greater ΔTCP value was observed in GTVn and CTVn. CTV had the largest TCP reduction.

**Fig. 7:**
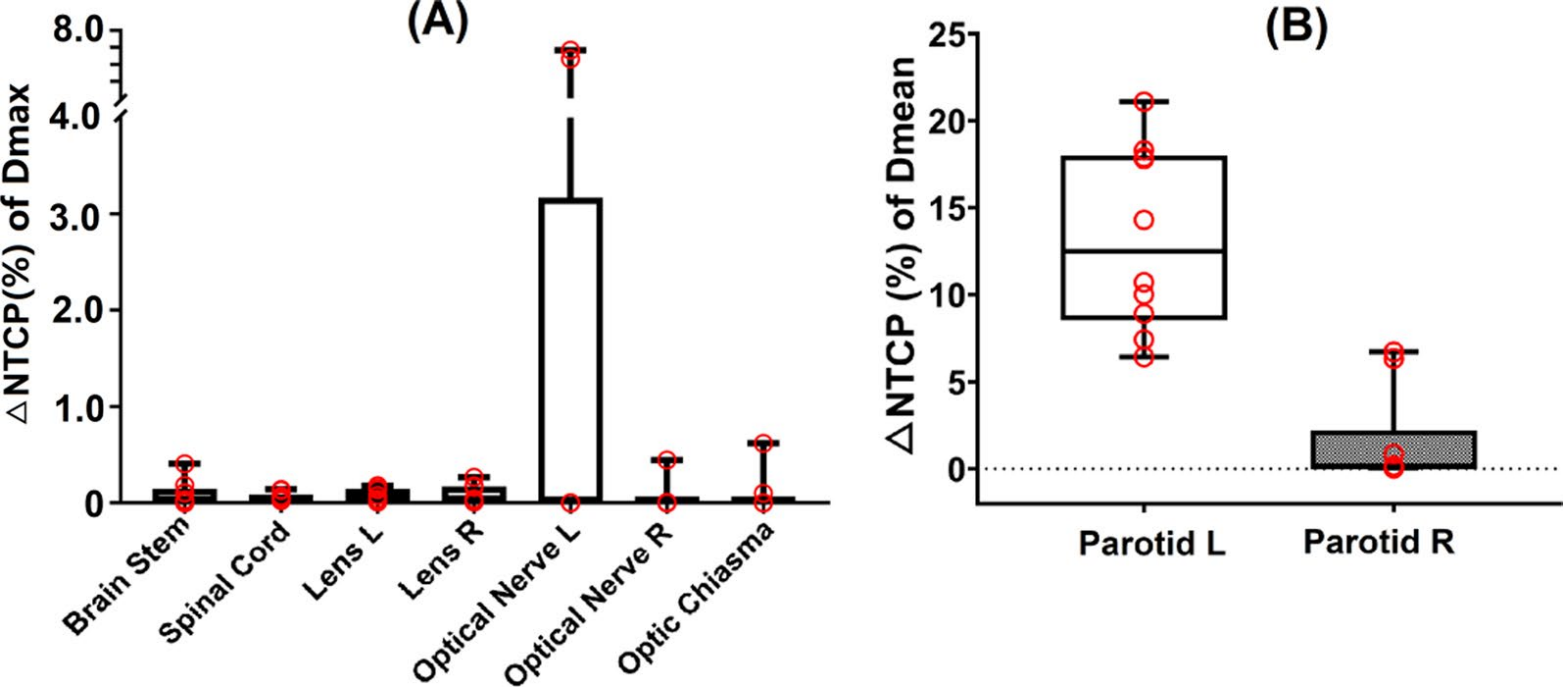
Box plot showed the ΔNTCP of OARs due to set-up uncertainties. The ΔNTCP was the mean reduction of NTCP

We performed NTCP modeling analysis to evaluate the dose variation of OARs (Fig. 7). The NTCP reduction (ΔNTCP) was obtained as the mean absolute value of the minimum value subtracted from the maximum value. The average ΔNTCP of bilateral parotids reached 6.17% (left) and 7.70% (right) (Fig. 7). No significant biological dose changes were found in OARs.

## Discussion

VMAT plans exhibited strong sensitivity to geometric deviation PTVp and PTVn with large ΔD_98%_ and ΔD_95%_. In photon radiotherapy, the CTV-to-PTV margin method was adopted based on the Van Herk margin formula [16] in the margin-based treatment planning, to ensure the dose coverage of CTV by blurring dose distribution induced by systematic setup errors. Although the CTV-to-PTV margin increased robustness in CTVp and CTVn, the ΔD_98%_ of CTVp and CTVn reached 1.12 Gy and 1.39 Gy. The ΔD_98%_ of GTVp and GTVn reached 0.56 Gy and 0.64 Gy. Similarly, considerable dose deviations were observed in D_95%_ of CTVp, CTVn, PTVp, and PTVn. Although the margin method effectively improved the plan's robustness by reducing sensitivity to the uncertainties, high risk remains. The dose variation of D_95%_ and D_98%_ in PTVs could reach a maximum of 6 Gy. The maximum difference of D_95%_ and D_98%_ in CTVs and GTVs could reach a maximum of 2.81 Gy. The maximum difference of D_mean_ of PTVs could reach 1.5 Gy. The study of Dupic [17] indicated that the GTV D_98%_ is a strong reproducible significant predictive factor of local control for the brain. A sufficient dose of GTVs should be rigidly reached. Zhao et al. [18] performed a retrospective study of a total of 1,092 patients with NSCLC of clinical-stage T1-T2 N0M0 who were treated with SABR. They recommended that both PTV D_95%_ and PTV_mean_ should be considered for plan optimization other than gross tumor volume. When the physical dose changed, the biological effect followed. The ΔTCP in GTVp and CTVp were respectively 0.4% and 0.3%. However, ΔTCP of GTVn and CTVn were 0.92% and 1.3% respectively. The CTV had the largest mean variation of ΔTCP (2.2%). Under dosage in the targets may result in the likelihood of tumor recurrence [19], for TCP predominately correlates with the minimum dose of tumor [13]. Plan robustness of photon radiotherapy should be taken into consideration.

Weak robustnesses and large dose variations were observed in the OARs in the vicinity locations of PTVs. In this study, the average ΔD_max_ of the brain stem and spinal cord reached 1.85 Gy and 1.51 Gy. Previous research reported that brain stem necrosis, MIR-based evidence of injury, or neurologic toxicities were related to photon radiotherapy [20–22]. Using conventional fractionation of 1.8–2 Gy/fraction to the full-thickness cord, the estimated risk of myelopathy is < 1% and < 10% at 54 Gy and 61 Gy, respectively [23]. For bilateral optic nerves and chiasm, the average ΔD_max_ were 4.59 Gy, 5.00 Gy and 5.01 Gy. There is a shred of strong evidence that evidence radiation tolerance is increased with a reduction in the dose per fraction [14, 24]. In radiotherapy of NPC, the bilateral parotids are often under irradiation. Salivary dysfunction has been correlated to the mean parotid gland dose, with recovery occurring with time [25–27]. The average ΔNTCP of bilateral parotids reached 6.17% (left) and 7.70% (right), which sharply increased the risk of parotid gland dysfunction. The actual irradiation dose of vicinal OAR may be biased upwards due to the set-up uncertainty.

Based on the results in this study, it is not hard to notice the strong sensitivity of highly optimized VMAT plans to geometric deviations. This generates worries about the accuracy of treatment dose delivery. ‘Plan quality assessment’ had been proposed firstly by the 3rd Physics ESTRO Workshop in 2019. Plan quality could be understood as the clinical suitability of the delivered dose distribution that can be realistically expected from a treatment plan [4]. Plan quality depends on the plan robustness and complexity of the treatment plan.

Intricate anatomical structures, precise dose coverage, and optimal OARs sparing generated highly optimized VMAT plans in NPC radiotherapy. High-degree modulated radiotherapy techniques increased plan complexity, with modulation of machine parameters, such as gantry rotate speed, continuously varied dose rate, and position of MLC. A study by Hirashima [28] uses plan complexity and dosiomics features to predict the performance for gamma passing rate, indicating the correlation between plan complexity and the accuracy of treatment plan dose delivery. Many commercial TPSs now offer the possibility to control plan complexity, such as controlling the minimum size and monitor unit (MU) (Phillips Pinnacle, Amsterdam, the Netherlands), aperture shape controller (ASC) (Varian Eclipse, Palo Alto, CA, USA), and modulation factor (MF) (TomoTherapy, Accuray Incorporated, Sunnyvale, CA, USA). The balance should be reached between dosimetric improvement and dose delivery accuracy.

Plan robustness qualification was always considered in proton therapy to address sensitivity to uncertainties in treatment planning [29]. In photon RT, the CTV-to-PTV margin method had been adopted to assure dose coverage with uniform margin, instead of plan robustness qualification. However, the CTV-to-PTV margin method has limitations, such as relying on the so-called static dose cloud approximation. A phantom study conducted by Englesman et al. [30] observed a maximum decreased dose of 5% with respiratory motion uncertainty. Guerreiro [31] evaluated the robustness against inter-fraction anatomical changes between photon and proton dose distributions and found that daily anatomical changes proved to affect the target coverage of VMAT dose distributions to a higher extent. Our results indicated that CTV-to-PTV margin increased robustness of CTV and GTV, reduced but did not remove the risk of underdosage. This plan robustness quantification method could be adopted in highly optimized clinical treatment plans to make a more complete dose description.

Besides, the robustness optimization methods had been developed by incorporating uncertainty in plan optimization, for CTV should receive the prescribed dose depending on desired dose distribution and dose fall-off near the target rather than geometric margin [32]. Lowe et al. [33] believed robustness optimization was an effective method to reduce dose to normal tissues that would be unnecessarily irradiated with the CTV-to-PTV margin concept. Dosimetric consequences of uncertainty, such as equivalent uniform dose (EUD), TCP, and NTCP were also recommended.

Among the limitation of the study, it is important to highlight that the first 5 times set-up errors acquired from CBCT did not represent the actual set-up uncertainty, for the set-up error consisted of systematic and random errors. Additionally, the patient anatomy change and rotation have not been taken into account. As a possible solution, adaptive radiotherapy (ART) could help to solve this problem [34]. We aimed to simulate the scenarios introduced to set up uncertainties, and visualize the necessity of robustness quantification is highly optimized photon RT. Treatment plan robustness analysis provides a more complete description of the dose delivered in the presence of uncertainties, and may lead to future dosimetric studies with improved accuracy.

## Conclusions

VMAT plans had a strong sensitivity to set-up uncertainty in NPC radiotherapy, due to the high degree of modulation. We proposed an effective method to evaluate the plan robustness of VMAT plans. Plan robustness and complexity should be taken into account in photon radiotherapy techniques with high degree optimization. The robust optimization may have the potential and could be considered in complex plans with a reliable evaluation of long-term clinical outcomes.

## Data Availability

The datasets used and/or analyzed during the current study are available from the corresponding author on reasonable request.

## Abbreviations

PTV: Planning target volume
PTVp: Planning target volume of GTVnx
PTVn: Planning target volume of GTVnd
CTV: Clinical target volume
CTVp: Primary tumor sites and their invasion range
CTVn: The clinical target volume of GTVn
GTVp: The clinical target volume of GTVp
GTVn: Cervical metastatic lymph node
CBCT: Cone beam computed tomography
VMAT: Volumetric arc radiotherapy
IMRT: Intentively modulated radiotherapy
3D-CRT: 3-Dimension conformal radiotherapy
OAR: Organs at risk
TCP: Tumor control probability
NTCP: Normal tissues complication probability
